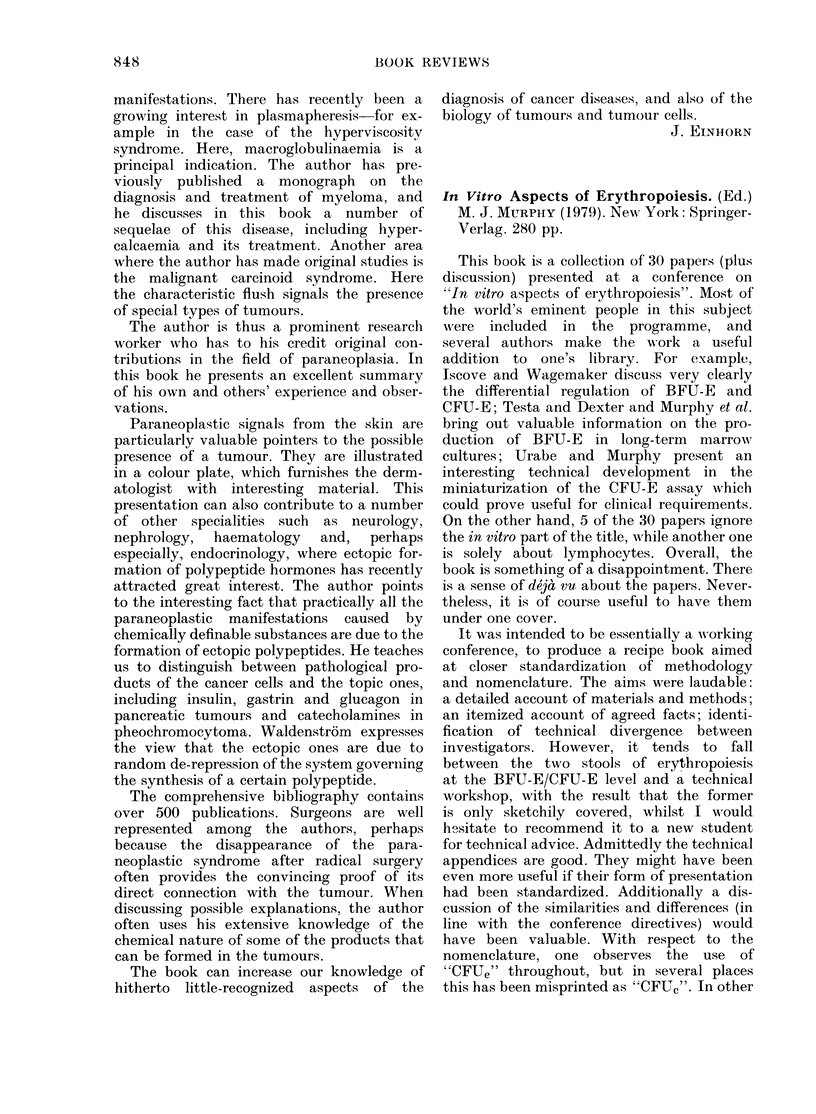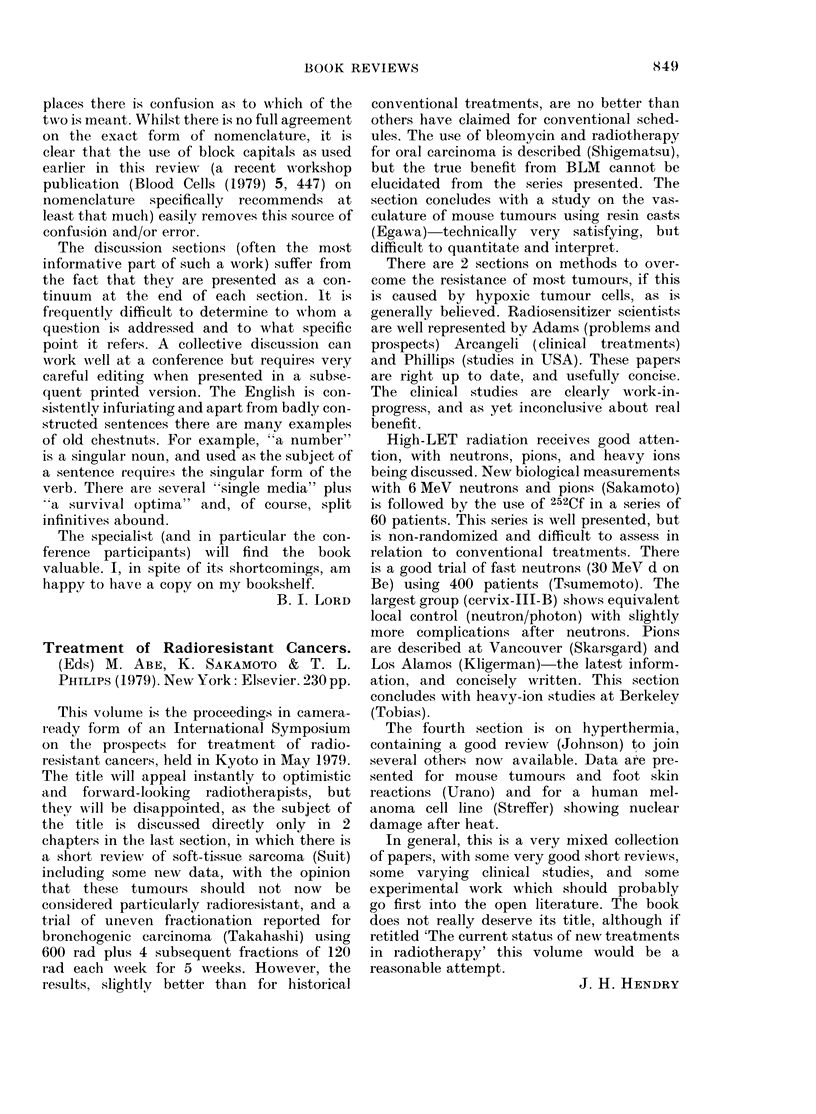# In Vitro Aspects of Erythropoiesis

**Published:** 1980-05

**Authors:** B. I. Lord


***In Vitro* Aspects of Erythropoiesis.** (Ed.) M. J. Murphy (1979). New York: Springer-Verlag. 280 pp.

This book is a collection of 30 papers (plus discussion) presented at a conference on "*In vitro* aspects of erythropoiesis". Most of the world's eminent people in this subject were included in the programme, and several authors make the work a useful addition to one's library. For example, Iscove and Wagemaker discuss very clearly the differential regulation of BFU-E and CFU-E; Testa and Dexter and Murphy *et al.* bring out valuable information on the production of BFU-E in long-term marrow cultures; Urabe and Murphy present an interesting technical development in the miniaturization of the CFU-E assay which could prove useful for clinical requirements. On the other hand, 5 of the 30 papers ignore the *in vitro* part of the title, while another one is solely about lymphocytes. Overall, the book is something of a disappointment. There is a sense of *déjà vu* about the papers. Nevertheless, it is of course useful to have them under one cover.

It was intended to be essentially a working conference, to produce a recipe book aimed at closer standardization of methodology and nomenclature. The aims were laudable: a detailed account of materials and methods; an itemized account of agreed facts; identification of technical divergence between investigators. However, it tends to fall between the two stools of erythropoiesis at the BFU-E/CFU-E level and a technical workshop, with the result that the former is only sketchily covered, whilst I would hesitate to recommend it to a new student for technical advice. Admittedly the technical appendices are good. They might have been even more useful if their form of presentation had been standardized. Additionally a discussion of the similarities and differences (in line with the conference directives) would have been valuable. With respect to the nomenclature, one observes the use of "$CFU_e$" throughout, but in several places this has been misprinted as "$CFU_c$". In other places there is confusion as to which of the two is meant. Whilst there is no full agreement on the exact form of nomenclature, it is clear that the use of block capitals as used earlier in this review (a recent workshop publication (Blood Cells (1979) **5**, 447) on nomenclature specifically recommends at least that much) easily removes this source of confusion and/or error.

The discussion sections (often the most informative part of such a work) suffer from the fact that they are presented as a continuum at the end of each section. It is frequently difficult to determine to whom a question is addressed and to what specific point it refers. A collective discussion can work well at a conference but requires very careful editing when presented in a subsequent printed version. The English is consistently infuriating and apart from badly constructed sentences there are many examples of old chestnuts. For example, "a number" is a singular noun, and used as the subject of a sentence requires the singular form of the verb. There are several "single media" plus "a survival optima" and, of course, split infinitives abound.

The specialist (and in particular the conference participants) will find the book valuable. I, in spite of its shortcomings, am happy to have a copy on my bookshelf.

B. I. LORD